# Noninvasive Follicular Thyroid Neoplasm with Papillary-Like Nuclear Features: An Evidence-Based Nomenclature Change

**DOI:** 10.1155/2017/1057252

**Published:** 2017-02-09

**Authors:** Rachel Jug, Xiaoyin Jiang

**Affiliations:** Duke Health, DUMC 3712, Durham, NC 27710, USA

## Abstract

A consensus panel recently used clinical evidence and pathologic parameters to rename noninvasive encapsulated follicular variant of papillary thyroid carcinoma to noninvasive follicular thyroid neoplasm with papillary-like nuclear features (NIFTP) to better reflect the indolent course of this tumor. NIFTP has stringent histopathologic diagnostic criteria established by the panel, including papillary-like nuclear features, and submission of the entire tumor capsule to exclude invasion. From a molecular standpoint, NIFTP is often characterized by RAS-type mutations, similar to other follicular-patterned lesions. While there has been prior evidence in the literature for the low malignant potential of these tumors, projects moving forward will help to independently reinforce the reliability of these criteria and nomenclature. With planned inclusion of NIFTP into the latest World Health Organization endocrine tumor classification scheme, this nomenclature shift provides a model for pathology efforts to refine diagnostic classifications to better guide treatment. In this review we discuss this nomenclature change and review the current literature.

## 1. Introduction

A group of experts in endocrine neoplasia were led by Dr. Nikiforov to formally assess the tumor previously known as “noninvasive encapsulated follicular variant of papillary thyroid carcinoma (FVPTC)” to develop diagnostic criteria and study prognosis. Based on the group's consensus findings, the new term “noninvasive follicular thyroid neoplasm with papillary-like nuclear features (NIFTP)” was announced at the meeting of the Endocrine Pathology Society in March 2015 [1], and the study was published in April 14, 2016, in an online publication of JAMA Oncology [2]. This name change made headlines in the medical community and mainstream media alike [3]. The terminology is planned for inclusion in the new World Health Organization classification system for endocrine tumors. The reclassification of NIFTP was undertaken to further characterize the clinical behavior of this lesion in an effort to prognosticate and manage patients appropriately to prevent potential overtreatment and reduce the burden to patients of a “cancer” diagnosis [4]. Pathological classification criteria accompanying the new name were developed [2, 5]. Beyond changing the classification of a small but significant proportion of thyroid cancers, this movement has provided a model for the reclassification of other low grade malignancies.

## 2. Clinical Presentation

Clinically, NIFTP presents similarly to most thyroid neoplasms by detection of a nodule on routine or focused physical examination or incidentally during unrelated diagnostic imaging. If NIFTP grows large enough, it can also potentially present by mass effect on surrounding structures, leading to dysphonia, globus sensation, or airway compromise [6].

## 3. Histopathological Findings

Grossly, NIFTP appears as a solid well-circumscribed or encapsulated nodule. The capsule may be thin and difficult to discern; however, a clear demarcation between the lesion and surrounding thyroid tissue will be readily appreciated. The cut surface is often paler than normal surrounding thyroid tissue and necrosis and hemorrhage are unlikely to be seen. In the NIFTP series studied by Nikiforov et al., the 109 tumors ranged in size from 1.1 to 9 cm [2].

**Figure 1:**
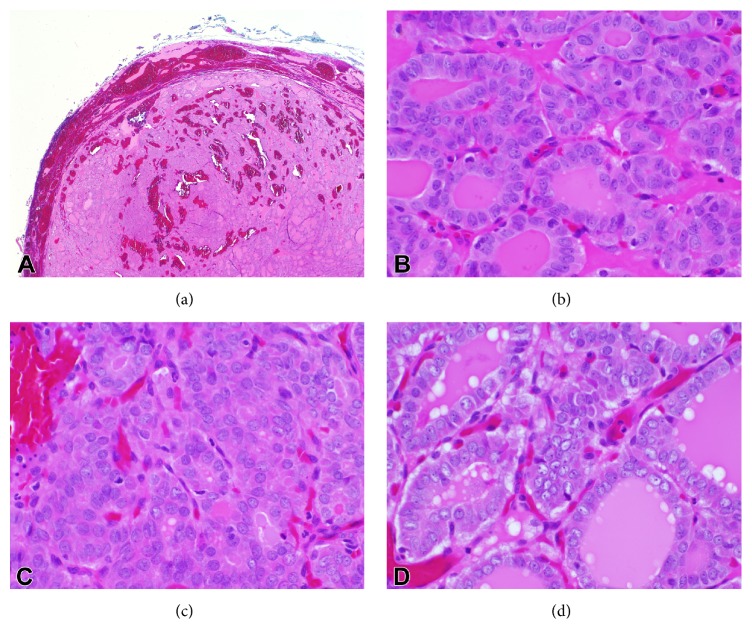
H&E images of NIFTP: (a) low-power (2x) view demonstrating a well-circumscribed lesion with follicular architecture. No vascular or capsular invasion is present. (b, c, d) High-power (40x) images demonstrating nuclear enlargement, crowding, optical clearing, and grooves.

The histological diagnosis of NIFTP is made using strict inclusion and exclusion criteria following submission of the entire tumor capsule for histology and careful examination of nuclear features, as well as overall architecture. The name NIFTP aptly conveys this lesion's prognosis and histologic features. Beginning with “noninvasive,” the capsule or tumor/normal interface must be entirely examined microscopically to rule out invasion as capsular or vascular invasion would exclude the tumor from a NIFTP classification (Figure 1(a)). Next, a follicular growth pattern defined as less than 1% papillae, <30% of the lesion composed of a solid, trabecular, or insular growth pattern, and a lack of psammoma bodies helps to classify this lesion as “follicular.” Acceptable follicular growth patterns include microfollicular, macrofollicular, and normofollicular with colloid. “Papillary-like nuclear features” must be present, with an overall qualifying score of 2-3 based upon a consensus grading system. Include 1 point each for the presence of either (1) nuclear enlargement/overlapping/crowding or elongation, (2) nuclear membrane irregularities, grooves, or pseudoinclusions, or (3) glassy nuclei/cleared chromatin with margination (Figures 1(b), 1(c), and 1(d)). Finally, there must be no tumor necrosis and mitoses must number less than 3 per 10 high-power fields (400x). These histological features are similar to those used formerly to diagnose the noninvasive encapsulated FVPTC, with the study authors proposing a nuclear scoring system with a 94.3% classification accuracy [2]. In addition, the current requirement of complete microscopic examination of the tumor-normal interface to make the diagnosis of NIFTP imposes standards for grossing of these specimens as well. Immunohistochemical stains apart from hematoxylin and eosin are not required for the diagnosis of NIFTP; however, HBME-1, galectin-3, and CK19 are positive in follicular-patterned tumors which could support a differential diagnosis including NIFTP and EFVPTC [7].

## 4. Impact on Cytopathology

**Figure 2:**
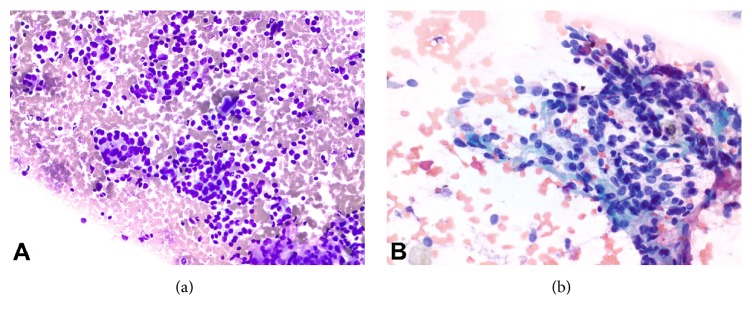
Cytology images of NIFTP: (a) high-power (20x, Diff-Quik) view demonstrating a cellular lesion with microfollicles. (b) High-power (40x, Papanicolaou stain) view showing nuclear elongation and grooves.

The introduction of the NIFTP nomenclature has posed a potential problem for cytopathologists. Often, the first specimens obtained from thyroid nodules for review by a pathologist are fine needle aspirates (FNA), on which evaluation of capsular invasion is not possible, and differentiating nuclear features of NIFTP and papillary thyroid carcinoma can be challenging. Many worry that making the diagnosis of PTC on cytopathology now carries the risk of a false-positive result in NIFTP. The Bethesda System for Reporting Thyroid Cytopathology (BSRTC) assigns a risk of malignancy to each of the possible diagnostic categories into which biopsies are placed by the pathologist. The creation of NIFTP has introduced a shift to the currently described risk of malignancy for each of these categories. Early studies seem to indicate that the risk of false-positive for a Bethesda VI diagnosis is low, with most NIFTPs being classified on preoperative FNA into indeterminate categories. Strickland et al. performed a single institution study correlating FNA biopsies to surgical specimens (655-patient cohort) and found NIFTP was most often classified into the indeterminate categories of the BSRTC (including atypia of undetermined significance/follicular lesion of undetermined significance, follicular neoplasm, and suspicious for malignancy) and thus correspondingly significantly lowered the risk of malignancy associated with each of these indeterminate categories. Most significantly, the risk of malignancy in the suspicious for malignancy category decreased by 41.5% [8]. Faquin et al. performed a multi-institutional group study with a cohort of 6943 thyroid lesions biopsied by FNA with 1827 surgical specimens available for diagnostic correlation and similarly found NIFTP lesions were assigned the three indeterminate diagnostic categories of the BSRTC and likewise lowered the associated risk of malignancy in each category. Most significantly, the suspicious for malignancy category had a decreased rate of malignancy by 17.6–23.4% with the addition of NIFTP lesions [9]. Similarly, Maletta et al. reviewed the cytology of 96 histologically proven NIFTP samples and they were proportionately assigned to the following Bethesda categories: 56% Bethesda IV, 27% Bethesda V, 15% Bethesda III, and 2% Bethesda VI [10]. These findings elucidate the need to revisit guidelines for surgical management since they are currently based on the risk of malignancy associated with BSRTC diagnostic categories that do not take NIFTP into account. On FNA specimens with nuclear features of PTC and/or follicular architecture, NIFTP is now on the differential diagnosis, just as was EFVPTC [11] (Figures 2(a) and 2(b)). Overall, the evaluation of NIFTP lesions and EFVPTC by FNA has proven difficult with a 29% false-positive rate and 9–58% true predictive value on preoperative assessment of follicular variant tumors [7].

## 5. Molecular Findings in NIFTP

Driving clonal alterations have been found in NIFTP tumors, though to date data is limited on the specific entity of NIFTP as such [2]. The genetic alterations responsible for NIFTP include RAS oncogene alterations, similar to those driving follicular adenoma and follicular carcinoma tumors, in contrast to the BRAF oncogenes mutated in the classic papillary thyroid cancer pathway [2]. In the literature on encapsulated follicular variant of PTC, Rivera et al. demonstrated genotypic abnormalities in 11 of 28 encapsulated FVPTC tumors of which 26% of the invasive tumors and 0% of noninvasive tumors harbored a BRAF mutation. In comparison, 36% of encapsulated FVPTC harbored RAS mutations in contrast to 10% of invasive FVPTC tumors [12]. Howitt et al. observed a similar rate (46%) of RAS mutations in a series of partially encapsulated or well-circumscribed FVPTC tumors [13]. Eszlinger et al. demonstrated BRAF mutations in 50% of [invasive] FVPTC [14]. These findings support the theory that NIFTP may be genetically distinct from classical PTC or the more aggressive [infiltrative] FVPTC.

Given the recent description of the entity, the diagnostic criteria for NIFTP do not include a characteristic mutation, much like other thyroid tumors. Molecular studies are much more likely to play a critical role in guiding management at the time of FNA of these lesions. The presence of RAS mutations may contribute supportive evidence favoring a diagnosis of NIFTP, follicular adenoma, or follicular carcinoma, in contrast to BRAF mutations which are more commonly observed in association with classical papillary thyroid carcinoma [12–14]. The presence of a BRAF V600E mutation would not be compatible with NIFTP.

The utility, predictive value, and reporting schemes of currently available molecular tests will need to be reassessed in the era of NIFTP. Early work confirms that the shift in nomenclature will alter test specificity and overall rate of malignancy [8, 15–17]. Wong et al. reviewed a cohort of 63 cases that had been tested with the Afirma Gene Expression Classifier (GEC) in which FNA biopsies yielded diagnoses of atypia/follicular lesion of undetermined significance or suspicious for a follicular neoplasm and corresponding surgical resection specimens were reviewed. They found that NIFTP accounted for 64% of the carcinomas (“suspicious” test results) detected by the Afirma GEC [17]. Valderrabano et al. assessed the performance of the ThryoSeq v2 at their institution by retrospectively reviewing results on Bethesda category III and IV specimens and extrapolated their results to predict the impact on the overall rate of malignancy presuming NIFTPs as a benign diagnosis. They estimated that the overall rate of malignancy would drop from 24% to 15%, the negative predictive value would increase from 88% to 94%, and the positive predictive value would decrease from 50% to 34% overall in both indeterminate categories [18]. At our institution, we have reviewed results of molecular testing from thyroid nodules subsequently diagnosed as NIFTP on surgical resection and showed that all of the surgically confirmed NIFTPs showed molecular alterations on both the ThyroSeq and GEC. Expectedly, all of the NIFTP cases were called Bethesda category III or IV on cytology and all harbored RAS mutations [19]. More independent work is clearly needed in this area to better characterize molecular test performance under the new nomenclature.

## 6. Differential Diagnosis

The main differential diagnosis for NIFTP is invasive encapsulated FVPTC as this lesion shares the well-circumscribed, often encapsulated structure, follicular architecture and papillary-like nuclear features with NIFTP and differs only with as much as a single focus of capsular or vascular invasion. Invasive encapsulated FVPTC harbors similar RAS-type mutations as NIFTP, follicular adenoma, and follicular carcinoma. In order to differentiate these diagnoses, the tumor capsule must be entirely submitted for microscopic examination to exclude invasion in the case of NIFTP [12].

Follicular adenoma is another entity that shares a similar morphological appearance and molecular genotype to NIFTP [4]. Follicular adenomas are well-circumscribed, partially, or completely encapsulated and composed of follicles that can be arranged in various growth patterns (microfollicular, macrofollicular, trabecular, etc.) and lack nuclear features of papillary thyroid carcinoma [20]. The establishment of a clear scheme for nuclear diagnostic criteria for NIFTP was helpful to attempt to address the considerable interobserver variability when pathologists were tasked with diagnosing FVPTC [21]. With a 94.3% classification accuracy reported for NIFTP using the nuclear scoring system, differentiating between NIFTP and follicular adenomas based on nuclear features may become more reproducible [2].

## 7. Prognosis and Treatment

While there is little in the literature assessing clinical outcome in NIFTP by the NIFTP name, there has been much in the literature describing the indolent clinical characteristics of EFVPTC without invasion [8, 9]. Ghossein conducted a clinicopathological study of 78 patients with follicular variant of papillary thyroid carcinoma at Memorial Sloan Kettering and found no recurrences or metastases after 11 years in all 42 patients with noninvasive encapsulated FVPTC. In contrast, 6% of participants with invasive FVPTC experienced adverse outcomes [22]. The seemingly benign behavior of the lesion was observed, but the evidence and nomenclature were not in place to appropriately label and simultaneously prognosticate it as various names were proposed, including tumor of uncertain malignant potential or multifocal papillary thyroid carcinoma [22]. Nikiforov et al. clarified the confusion surrounding classification of this lesion by demonstrating the excellent prognosis associated with the diagnosis of NIFTP. After 10–26 years of follow-up, all 109 patients in the NIFTP group were alive without evidence of disease recurrence. In contrast, 12% of 101 patients in the encapsulated FVPTC with invasion experienced an adverse event within 1–18 years of follow-up [2]. In a series of 94 patients with encapsulated follicular variant of papillary thyroid carcinoma, Thompson found no evidence of recurrence or lymph node metastasis (42 patients underwent lymph node sampling) after a median follow-up time of 11.8 years [4].

Early reports suggest that nodal metastasis is a rare event in NIFTP, with one series presented at the 2016 American Thyroid Association meeting demonstrating no NIFTPs with nodal metastases [23] and another demonstrating a low rate (2.2%) of metastasis of NIFTPs at presentation [24]. Hahn et al. identified central nodal metastasis in 1 of 34 cases of NIFTP in their series [25]. It is unclear whether these cases of NIFTP with potential metastasis all underwent complete histopathologic evaluation of the entire tumor capsule, which is currently required to make a definite determination of NIFTP. Another limitation is the potential for occult microcarcinoma metastasis from another area of the thyroid giving rise to metastases then attributed to the NIFTP. We have reported in our series a case of potential lymph node metastasis from an entirely submitted NIFTP to a central lymph node [19], in which the remainder of the thyroid was submitted entirely, excluding the possibility of an occult microcarcinoma. This case suggests that caution and further study are required to better characterize the behavior of NIFTPs.

Prior to the reclassification of NIFTP, there was a lack of evidence to support conservative management (lobectomy alone) of noninvasive encapsulated FVPTC, and if the lesion measured greater than 1.5 cm, many physicians recommended completion thyroidectomy with subsequent radioactive iodine therapy and extensive follow-up, similar to conventional papillary thyroid carcinoma [22]. In addition to demonstrating the indolent clinical course of the pathologically defined NIFTP, Nikiforov et al. found that none of the 101 patients classified in the NIFTP group received radioactive iodine and 67 of them were treated solely by lobectomy [2]. Subsequently, Thompson of the Southern California Permanente Medical Group studied a series of 101 cases of encapsulated FVPTC, including 24 invasive and 77 noninvasive tumors, and of the 69 patients managed with surgery alone, there was an 11.8-year event-free median survival [4].

The American Thyroid Association strongly recommends follow-up of differentiated thyroid cancer by means of serial serum thyroglobulin measurement, serum thyroid stimulating hormone (in patients on thyroid hormone therapy), cervical/neck ultrasonography, and other tests depending on the clinical scenario [26]. Under the name of noninvasive encapsulated FVPTC, NIFTP was traditionally managed this way postoperatively due to a lack of evidence to support a more conservative approach. According to Thompson, NIFTP would fit into the lowest point on the “Risk Continuum” which corresponds to the treatment recommendation of surgical lobectomy without radiotherapy, a prophylactic central neck lymph node staging, or staging since NIFTP is not considered malignant [7]. This nomenclature shift has the potential to begin to reduce the burdens, with more studies adding to the evidence for these tumors' good prognosis. The diagnosis of cancer, treatment, and follow-up places financial strain on healthcare systems and patients and causes both financial and emotional consequences for patients.

## 8. Conclusions

The rigorous evidence-based efforts made by the expert panel convened to reclassify noninvasive encapsulated FVPTC to NIFTP have paved the way for reframing our approach to diagnosis and treatment of indolent thyroid lesions. They provided clear criteria for diagnosis and supporting evidence with long-term follow-up demonstrating benign behavior in the population they studied [2]. NIFTP, at least in initial reports, shares molecular alterations with follicular lesions of the thyroid (RAS oncogenes) as opposed to those found in PTC (BRAF oncogenes) [2, 12–14]. The establishment of NIFTP has prognostic implications that will modify clinical decision-making and the risk of malignancy associated with BSRTC diagnostic categories on thyroid FNA specimens. Early data suggest the majority of NIFTP fall into the indeterminate categories, thus while the concern of NIFTP leading to false-positive Bethesda VI diagnoses is a valid one, it does not appear to be a frequent occurrence [8, 9]. With widespread acceptance by inclusion of NIFTP in the latest World Health Organization endocrine tumor classification scheme, more research moving forward will be useful to better characterize the molecular findings of this lesion and build the literature on the long-term follow-up of these tumors. Large-scale population based studies are needed to further characterize the behavior of these tumors since small retrospective studies have demonstrated a possible low risk of nodal and distant metastatic disease in tumors classified as NIFTP [24].
